# A Prospective Intervention Trial on Tailored Radiofrequency Ablation of Uterine Myomas

**DOI:** 10.3390/medicina56030122

**Published:** 2020-03-12

**Authors:** Alessandro Fasciani, Giovanni Turtulici, Giacomo Siri, Simone Ferrero, Rodolfo Sirito

**Affiliations:** 1 Division of Gynaecology, International Evangelical Hospital, 16122 Genoa, Italy; alessandro.fasciani@oeige.org (A.F.); rodolfo.sirito@fastwebnet.it (R.S.); 2 Division of Radiology, International Evangelical Hospital, 16122 Genoa, Italy; 3 Department of Mathematics, University of Genoa, 16146 Genoa, Italy; siri.giacomo03@gmail.com; 4 Academic Unit of Obstetrics and Gynecology, IRCCS Ospedale Policlinico San Martino, 16132 Genoa, Italy; 5 Department of Neurosciences, Rehabilitation, Ophthalmology, Genetics, Maternal and Child Health (DiNOGMI), University of Genoa, 16121 Genoa, Italy

**Keywords:** Uterine fibroids, radiofrequency myolisis, minimally invasive surgery, ultrasonography, laparoscopy, hysteroscopy

## Abstract

*Background and Objective:* Investigating the use of radiofrequency myolysis (RFM) for the treatment of fibroids through less invasive access by combining transvaginal ultrasound, hysteroscopy and laparoscopy. *Materials and Methods:* Fifty-four premenopausal women with 106 symptomatic uterine myomas. Patients underwent RFM in three ways: Vaginal Ultrasound-guided RFM (VU-RFM), Laparoscopic RFM (L-RFM) and Hysteroscopic-RFM (H-RFM). The mean patient age was 43 years; 52 symptomatic uterine myomas were subserosal, 44 intramural and 10 submucosal. The outcomes evaluated at 1 and 12 months after RFM were myoma size (volume-diameter), “Uterine Fibroid Symptom and Quality of Life (UFS-QOL)” questionnaire and a 10-point Visual Analogue Scale (VAS). The therapy was completed with a single ablation in all patients, no complication was registered. The average number of fibroids treated per intervention was two with the use of different accesses: 64/106 VU-RFMs (60.4%), 32/106 L-RFMs (30.2%) and 10/106 H-RFMs (9.4%). *Results:* Volume and diameter of fibroids were significantly reduced by, respectively, 51.3% and 20.1% in the first 30 days post-intervention (*p* < 0.001) up to a maximum of 73.5% and 37.1% after the second follow-up visit at 12 months (*p* < 0.001). A similar trend was shown in terms of disability with a progressive and significant reduction of symptoms (menorrhagia, dysmenorrhea, dyspareunia and pollakiuria) demonstrated by percentage variation of UFS-QOL Symptom Severity and VAS scores to −74.3% and −45.3% as well as −84.9% and −74.3%, respectively, at 1 and 12 months after RFM (*p* < 0.001). An overall improvement in the quality of life was also demonstrated by a significant increase in the UFS-QOL total score of +38.2% in the first 30 days post-intervention up to +44.9% after the second follow-up visit at 12 months (*p* < 0.001). The overall average surgery time of the RFM for each patient was 48 minutes, and the time to treat each fibroid by Vaginal Ultrasound-guided RFM (23 min) was found to be significantly less than those of laparoscopy or hysteroscopy (respectively 35 and 34 min) (*p* < 0.05). An electromagnetic virtual needle tracking system (VNTS) was successfully tested during the RFM procedures, and real-time contrast-enhanced ultrasound (CEUS) has proven to be effective in determining the duration of myolysis through the identification of eventual residual areas of enhancement within the fibroids. *Conclusion:* Radiofrequency can be considered a minimally invasive and safe procedure for the treatment of uterine myomas through the customization and possible combination of transvaginal, laparoscopic or hysteroscopic accesses. The standardization of the ablation technique with pre-intervention biopsy and new technologies such as VNTS and CEUS spares healthy uterine tissue and may change the future management of symptomatic uterine fibroids.

## 1. Introduction

Uterine fibroids, benign tumors affecting up to three women out of four in the premenopausal age, are often the cause of abnormal uterine bleeding, pelvic pressure, sub-fertility and dyspareunia. Their impact on the health-related quality of life (HRQOL) is well reported in the literature [1]. Because of their incidence and symptomatology, uterine fibroids are an important social and health burden and are the main cause of gynecological hospitalization accounting for approximately 80% of hysterectomies [2]. Even other treatment options (the removal of myomas and uterine artery embolization) do not avoid hospitalization with overnight stay and general anesthesia, thus determining a delay in the patient’s return to normal daily life and potential severe adverse events [3]. Despite this, there is often no clear agreement among gynecologists about which treatment is the most appropriate. Similar cases in terms of symptoms and types of myomas may be treated expectantly by some physicians and surgically by others [4] since comparative effectiveness of data on uterine fibroid treatments are still lacking [5]. 

This situation clearly indicates that there is a prevalence of invasive approaches; therefore, there is an urgent need for non-invasive fibroid therapy that possibly saves the uterus and can also treat intramural myomas without incisions (like the submucosal fibroids treated by hysteroscopy) [6]. Ideally, a conservative management for uterine fibroids should be safe, treat symptoms, decrease the volume of myomas, preserve fertility and have long-term quality of life effects [7].

In the last few years new mini-invasive techniques have been developed for the treatment of uterine fibroids that have improved women’s quality of life [8,9]. These incisionless procedures use various forms of energy to heat and ablate uterine fibroids like radiofrequency, focused ultrasound and microwaves. Recently MR-guided focused ultrasound (MRgFUS), another modality of hyperthermic ablation, has been approved by the US Food and Drug Administration as a validated treatment for fibroids in women seeking pregnancy [6].

Radiofrequency myolysis (RFM) is the latest uterine-conserving technique, generates thermal effects inside the myoma (60–80 °C), and it results in three distinct therapeutic histological changes: (1) the death of tissue cells from coagulation, (2) formation of vascular thrombosis in the blood vessels that supply the myoma with consequent its ischemic necrosis/atrophy and (3) inactivation of hormonal receptors within the myoma that prevents tumor tissue from growing [10].

Although initial data has already indicated that RFM is the most promising of the minimally invasive treatments of symptomatic fibroids [10] and adenomyomas [11], its clinical use still has not been standardized with protocols and uniform patient selection criteria. The excessive differences in the use of RFM amongst surgeons, both regarding indications and access, have led to discordant efficacy data with consequent controversy surrounding its application in gynecology [10].

Given that the RF ablation of solid tumors of the liver and other organs is successfully used as a standard approach [12,13,14], we decided to transfer this technology to the treatment of uterine fibroids. Indeed, in order to achieve the optimization of radiofrequency myolysis, we improved the procedure by inserting three innovative elements: (1) application of a virtual needle tracking system to follow the RF electrode during the ablation session [15,16], (2) real-time monitoring of RF ablation by contrast-enhanced ultrasound [17,18] and (3) systematic biopsy of the lesions before electro-coagulation [19]. 

The aim of our work was to investigate the use of RFM for the treatment of uterine fibroids through less invasive access by combining trans-vaginal ultrasound, hysteroscopy and laparoscopy. The access to ablate each individual fibroid was decided depending on the shortest possible route to reach its center. In this alternative way of performing RFM, the access route is chosen on the basis of the localization of the fibroid rather than on the experience of the operator. A second objective of this customization process was to minimize risks and complications through the use of safety elements such as the visualization of myoma by contrast-enhanced ultrasound (CEUS) and virtual needle tracking systems beyond the systematic biopsy of all fibroids treated with RFM.

## 2. Materials and Methods

### 2.1. Enrolment Criteria 

In this single-center, self-controlled study, 54 patients with 106 fibroids were recruited from March 2017 to March 2018. The enrolled patients were counselled about the potential risks, benefits or possible alternative treatments to RFM, and all women provided written informed consent. The Local Institutional Review Board approved this observational prospective study (CER Liguria N. 295, date: 8 April in 2019), which was also registered at ClinicalTrials.gov (identifier: NCT04091529). No conflicts of interest are associated with this publication, and there has been no significant financial support for this work that could have influenced its outcome.

The study included premenopausal women who had myomas with diameters ranging between 1.2 and 7.7 cm. The participants had previously declined hysterectomy or laparoscopic myomectomy indicated to them for symptoms caused by fibroids. Patients ranged in age between 30 and 62 years (mean of 43 years) and were all Italian with a desire to solve the symptoms in a minimally invasive way. All patients underwent a transvaginal ultrasonography, and there was no suspicion of malignancy. According to the International Federation of Gynecologists and Obstetricians (FIGO) classification, fibroids were classified into three groups: (1) submucosal (SM) (G2 type, with intramural extension ≥50%), (2) intramural (IM) (G3/G4 types) and (3) subserosal (SS) (G5/G6 types) [20]; respectively 10, 44 and 52 cases. Participants with intracavitary (G0/G1 types) or subserosal-pedunculated fibroids/(G7 types), genital malignancy, cervical dysplasias, pelvic infection/adhesions, severe systemic diseases, pregnancy and endometriosis/adenomyosis were excluded from the study. Patients who took gonadotropin-releasing hormone therapy or acetate ulipristal within 6 months were also not admitted to the RFM study.

Patient subjective myoma-related symptoms were assessed using the Uterine Fibroid Symptom and Quality of Life questionnaire [1], and a 10-point visual analogical scale was used for each reported symptom (menorrhagia, dysmenorrhea, urinary frequency and dyspareunia) (Supplementary Document 1).

### 2.2. Instruments

**Figure 1:**
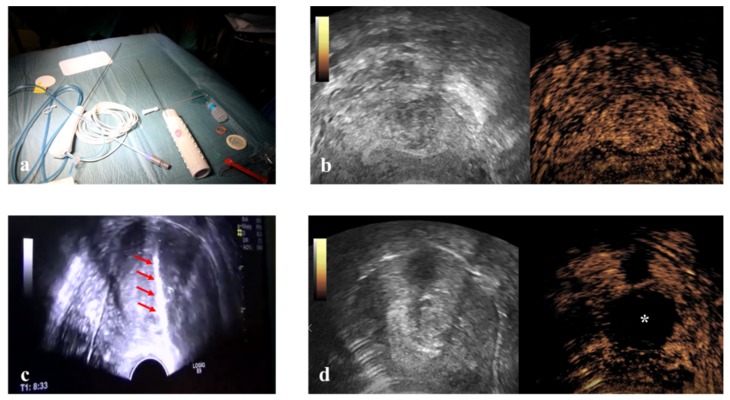
(**a**) 35 cm long 17 Gauge radiofrequency electrode at the left of the table and an 18 gauge disposable core biopsy instrument at the right. (**b**) Contrast-enhanced ultrasound (CEUS) evaluation before radiofrequency ablation of a fibroid. (**c**) Hyperechogenic line within the myoma viewable during the biopsy (arrowheads). (**d**) CEUS evaluation after radiofrequency (RF) ablation demonstrates no residual contrast enhancement within the fibroid (asterisk).

This prospective study enrolled 54 consecutive patients. RFM was performed in three ways: Vaginal Ultrasound-guided RFM (VU-RFM), Laparoscopic RFM (L-RFM) and Hysteroscopic-RFM (H-RFM). These three methods of myolysis were performed both separately as an exclusive type of surgery and in a combined approach when the localization of the myomas required to use the same electrode through different access routes. The ablation device consisted of an RF energy generator (STARmed, VIVA RF generator VRS01, Gyeonggi-do, Korea 10355) and an electric pump for the continuous cooling of the electrode tip. The RF generator to which the electrode (STARmed Gyeonggi-do, Korea 10355) was connected simultaneously displayed the temperature of the electrode tip, the tissue impedance characteristics, the power and the ablation time. A 35 cm long 17G internally cooled electrode with an exposed tip of 10 mm or a variable exposed tip was used (Figure 1). 

Pre-treatment evaluation was performed for all patients with conventional trans-abdominal and transvaginal (TV) ultrasound using an ultrasound machine equipped with a 3–5 MHz convex probe and a 1–6 MHz trans-vaginal probe (Logiq E9, GE Healthcare, Milwaukee, WI, USA). Contrast-enhanced ultrasound (CEUS) evaluation was also performed for all patients after the intra-venous injection of SonoVue micro-bubbles contrast (4.8 mL; Bracco, Milan) to evaluate the vascularity of myomas (Figure 1). The micro-bubble contrast agent was mixed with 5 mL of normal saline solution and was administered via rapid bolus into the median cubital vein, followed immediately by 5 mL of normal saline solution.

Immediately before ablation, all patients were subject to a US-guided core-needle biopsy of all fibroids to be treated by 18 Gauge Bard Max-Core disposable core biopsy instruments (#MC1820, Bard Limited Crawley, UK) (Figure 1). At least two biopsies were taken for each myoma, but the number of biopsies changed according to the size of the fibroid (one biopsy per centimeter of fibroid) so that all areas of the fibroid could be evaluated. All tissue samples were sent to the Institute of Pathology for routine analysis, and all samples were classified as representative by pathology.

### 2.3. Pre-ablation Preparation

Before ablation, all patients were admitted for presurgical analysis routinely required by the day surgery unit (blood work, urine tests and electrocardiography). A US scan was performed within 10 days before treatment to obtain an accurate assessment of the number, dimensions and location of the uterine fibroids in order to establish the appropriate approach route for the RF electrode. The mean diameter and volume of each fibroid were calculated by ultrasound using the following formulas: Mean diameter = (Length + Width + Height)/3, and Volume = 4/3 × π × r3, where r is the mean radius at ultrasound (mean diameter/2).

### 2.4. Therapy

**Figure 2:**
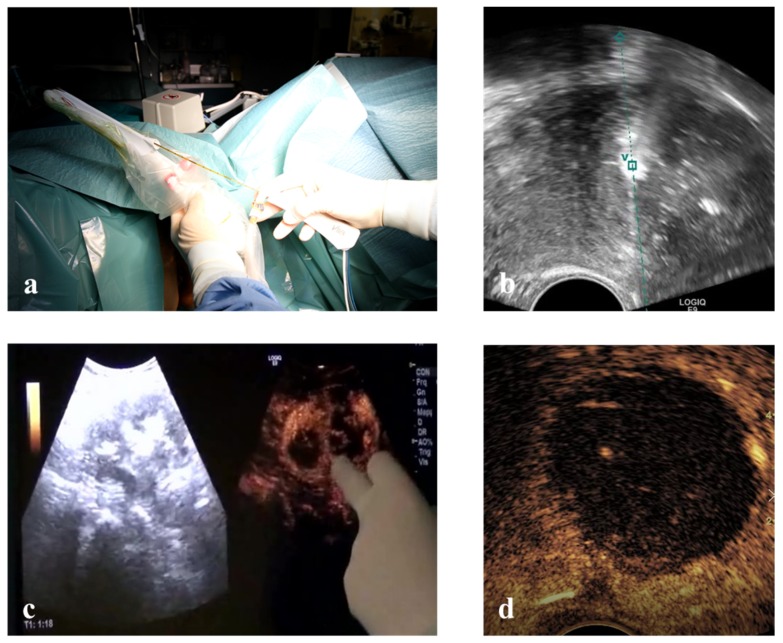
(**a**) Electromagnetic sensors attached to the transvaginal probe and to RF electrode connect to the low-field magnetic generator (cubic box) to provide a 3D operating volume navigation. (**b**) Virtual needle tracking: the path of the electrode is the dashed line and the hyper-echogenicity of RF ablation is precisely behind the active tip located with a green V. (**c**) Intra-operative CEUS shows residual areas within the myoma still to be treated. (**d**) Echo-enhanced area after a complete RF ablation of a fibroid.

Vaginal ultrasound-guided RFM: unconscious sedation or spinal anesthesia and dorsal position were performed on an outpatient basis after a TV ultrasound re-evaluation of the patient. Before ablation the VT track system (VirtuTRAX, CIVCO Medical Solutions, Coralville, IA, USA) was set and used together with the volume navigation system (V-Nav, LOGIQ E9, GE Healthcare, Milwaukee, WI, USA). This platform has a Global Positioning System (GPS) tracking capability to generate a 3D operating volume around the patient through the connection between three electromagnetic position sensors (2 attached to the US probe and one secured on the shaft of the RF electrode) and the position-sensing unit (Figure 2). The 10 mm active tip was then synchronized with the tracking device by a manual input of the RF electrode length, thus locating the exact position of the RF electrode and projecting its path on the US monitor during the procedure. More specifically, this system is able to guarantee an in-plane approach by displaying a green line on the B-mode when the axis of the electrode fully matches the US scan plane with the projection of the active tip marked as a square, which becomes smaller as the RF electrode approaches the image plane. From that moment onwards, the path of the RF electrode is represented by a dashed line, the prospective RF electrode path is represented by a dotted line and its active tip is located with a green V [21] (Figure 2).

**Figure 3:**
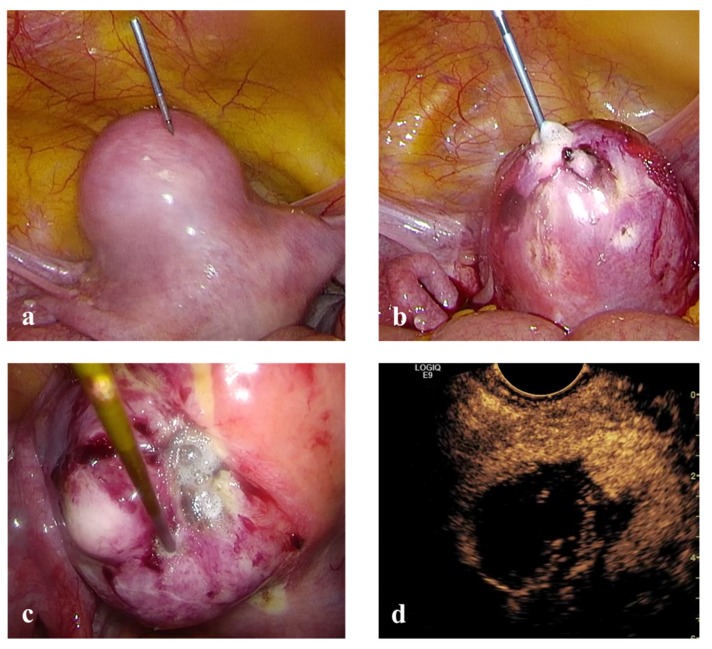
(**a**) RF electrode inserted directly into the abdomen without the aid of any trocar. (**b**) and (**c**) RF ablation of uterine myomas under laparoscopic control. (**d**) Intra-operative trans-vaginal CEUS during Laparoscopic RFM.

Laparoscopic RFM: general anesthesia, dorsal position, standard laparoscopic instrumentation and transvaginal ultrasonography for intra-uterine needle guidance (Figure 3).

**Figure 4:**
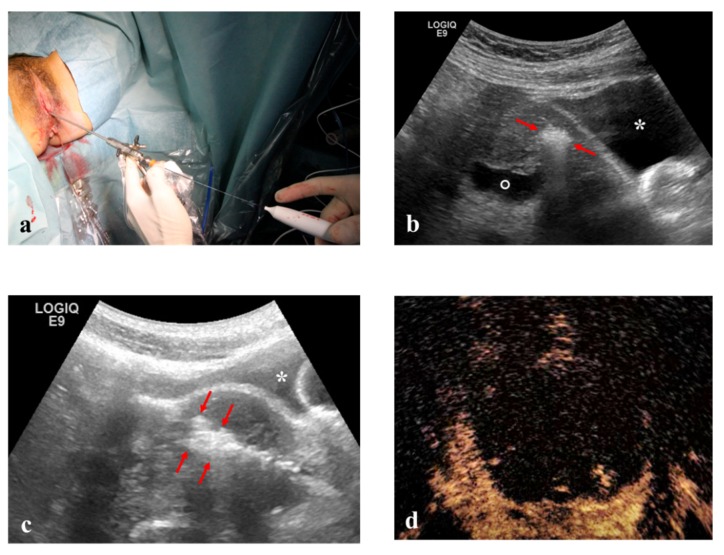
(**a**) RF electrode inserted into 5 Fr. working channel of a 3.8 mm hysteroscope. (**b**) and (**c**) Intra-operative TA ultrasound shows hyper-echogenicity of RF ablation of an anterior myoma (arrowheads) between the bladder (asterisk) and the endometrial cavity (empty point). (**d**) Echo-enhanced area after complete RF ablation of the fibroid.

Hysteroscopic RFM: unconscious sedation or spinal anesthesia and dorsal position were performed on an outpatient basis. Standard hysteroscopic instrumentation (Outer diameter 3.8 mm and straight 5 French (Fr) working channel, Wolf Medical Instruments, Germany) (Figure 4), and trans-abdominal ultrasonography for intra-uterine needle guidance.

Once the safest path to the target fibroid was identified both via ultrasound (TV or TA) and optically (L-RFMs and H-RFMs), the electrode was appropriately placed into the target fibroid under US real-time guidance and VT [21]. Ablation was performed with the ‘‘moving shot and stepping shot’’ technique [22], whereby the RF electrode was inserted in the distal part of the fibroid and then moved backward in steps of 5–10 s. The RF generator operated at 480 kHz with a maximum power of 200 W and at a temperature ranging from 40 to 99 °C [23]. The chosen working temperature within the fibroids was 85 °C with an automatic adjustment of the power by the device to maintain the selected temperature. Since the RF ablation time depends not only on the temperature but also on the impedance of the tissues, the tip of the electrode was cooled through a system of recirculation of cold saline solution in order to avoid the phenomenon of carbonization. Continuous US was used to monitor the procedure. The total time of ablation was determined based on increased echogenicity and continued until the echo-enhanced area reached 80–90% of the whole fibroid. At the end of ablation CEUS was performed in order to detect any eventual remaining area on enhancement (Figure 2) [21].

After the procedure, the patients were monitored for half a day, and then, if no complication was present, they were discharged.

### 2.5. Effectiveness Assessment

The outcomes evaluated at 1 and 12 months after RFM were myoma dimensions (volume–diameter), Uterine Fibroid Symptom and Quality of Life (UFS-QOL) questionnaire with scores ranging from 0 to 100, where higher QOL scores mean a better health-related QOL [1], and a 10-point scale used for each reported symptom. Major complications as situations that required further interventions and/or hospitalization were also recorded. To minimize any inter-observer variations, ultrasonograms were assessed by the same trained sonographer-ultrasonologist (AF).

All the ultrasound studies and the surgical interventions were recorded with digital images, and any resulting CEUS video-clips were digitally recorded and analyzed by means of Q-Contrast software V.4.0. (Bracco, Milan, Italy, 20134).

### 2.6. Statistical Analysis

Patients and fibroids were described through mean (SD), range (min–max) and frequencies. A one-way ANOVA was performed to assess the time of intervention according to different methods. In order to evaluate a percentage change through time, the log transformation of the outcome was adopted. The multiple Linear Mixed Model, adjusted for age and total number of fibroids per patient, was used to obtain the estimates of the effect of the RFM on the different outcomes of interest (volume and diameter reduction, VAS, UFSQOL scores); this approach allowed us to consider the fact that repeated measurements on the same patient were correlated. A Wald test was adopted to evaluate the effect of time over the outcomes. All analyses were conducted using STATA (version 14.2, StataCorp., College Station, TX, USA) software. Two-tailed probabilities were reported, and a *p* value of 0.05 was used to define nominal statistical significance.

## 3. Results

Fifty-four women enrolled in the study were all symptomatic (70.4% menorrhagia, 59.3% dysmenorrhea, 29.6% dyspareunia and 11.1% pollakiuria) with UFS-QOL symptom severity, UFS-QOL total and VAS score, respectively, of 38.9 (11.8–75.0), 67.2 (35.3–90.5) and 4.4 (0–8). Patient characteristics are shown in Table 1.

Out of 106 treated fibroids, 52 were subserosal (49.1%), 44 intramural (41.5%) and 10 submucosal (9.4%). Myoma volumes ranged from 0.9 to 217.5 cm^3^ (mean 35.4 cm^3^), and diameters ranged from 1.2 to 7.7 cm (mean 3.44 cm). The details are reported in Table 1. Histological examination confirmed the diagnosis of benign uterine fibroids in all the biopsies.

Each patient was treated by single or combined-access: 64/106 VU-RFMs (60.4%), 32/106 L-RFMs (30.2%) and 10/106 H-RFMs (9.4%). In all patients, therapy was completed during a single surgical session, no complication was registered, and the median number of fibroids treated per intervention was 2.0 (1–4). The overall surgery time of the RFM for each patient was 48 min (25–95), and the time to treat each fibroid trans-vaginally (23 min) was found to be significantly less than those of laparoscopy or hysteroscopy (respectively 35 and 34 min) (*p* < 0.05). No major complications (bleeding/infection/perforation) occurred in the study, and only two patients were re-operated: the first because the myoma did not allow access to an oocyte retrieval and the second for persistence of symptoms despite the decrease of fibroids volume.

Volume and diameter of fibroids were significantly reduced by 51.3% (95% Confidence Interval 37.3–62.2) and 20.1% (95%CI 12.5–27.1), respectively, in the first 30 days post-intervention (*p* < 0.001) up to a maximum of 73.5% (95%CI 61.3–81.9) and 37.1% (95%CI 29.5–43.8) after the second follow-up visit at 12 months (*p* < 0.001) (Table 2).

A similar trend was shown in terms of disability with a progressive and significant reduction of symptoms demonstrated by percentage variation of UFS-QOL Symptom Severity and VAS scores to −74.3% (49.6–86.9) and −45.3% (26.0–59.5) as well as −84.9% (76.2–90.4) and −74.3% (63.1–82.1), respectively, at 1 and 12 months after RFM (*p* < 0.001) (Table 2). A general improvement in the quality of life was also confirmed by a significant increase in the UFS-QOL total score of +38.2% (19.9–59.2) in the first 30 days post-intervention up to +44.9% (17.1–79.3) after the second follow-up visit at 12 months (*p* < 0.001) (Table 2). At 12 months after RFM, all parameters were significantly improved over baseline scores.

## 4. Discussion

This paper describes the pioneering strategy of radiofrequency ablation with systematic biopsy of uterine fibroids using transvaginal, endo-cavitary or laparoscopic accesses supported by virtual-electrode tracking and real-time contrast-enhanced ultrasound. This study shows that the optimization and standardization of radiofrequency myolysis may provide women with uterine fibroids a minimally invasive treatment that alleviates symptoms, prevents hospitalization, provides a histological assessment, maintains uterus/fertility and can eventually be associated with other surgical intervention or medical treatments.

The approach of choosing the center of the myoma in a perpendicular direction and the use of shorter electrode length, thus sparing healthy uterine tissue, has led to shorter operating times and very positive results in clinical outcome. To the best of our knowledge, this is the first report in the literature in which the location/shape of each individual fibroid has been used to determine the radiofrequency ablation pathway rather than the surgeon’s preference or expertise, consequently offering different or combined approaches in the same patient and in a single surgical session. The combined approach of transvaginal ultrasound, hysteroscopy and laparoscopy has, as a matter of fact, made treatment of different types of myoma possible at the same time, with significant savings both in terms of patient’s psycho-physical well-being and health resources.

In our opinion it is important to underline that, when compared to the other two main alternative uterine-sparing techniques for fibroids, MRgFUS and uterine artery embolization (UAE), RFM does not require the use of ionizing radiation or magnetic resonance, can be easily combined with hysteroscopy and, in our protocol, is associated with the biopsy of each ablated myoma. Furthermore, the histopathological analysis should be considered a security safeguard in any conservative treatment of uterine fibroids, especially when performed as a “bridging therapy” in peri-menopausal symptomatic patients. Indeed, our model of RFM takes into consideration both the clear indication laid out by current guidelines to perform hysteroscopy in cases of abnormal uterine bleeding [20] and recent FDA warnings for the potential risk of uterine sarcomas. This is because an overlap in imaging presentation between atypical sarcomas and degenerated leiomyomas is known to be often misleading [24,25].

Fibromatosis in women seeking pregnancy or suffering infertility generates lesions that alter both the anatomy and function of the uterus by compromising the endometrial–myometrial blood supply and the local hormone milieu could impair gamete transport. Moreover, myomas cause obstetric complications such as pre-term delivery (<37 weeks), lower birthweight infants, primary cesarean section and breech presentation [26]. RFM might be considered in infertile women with myomas. Moreover, this technique avoids the negative effects of the UAE on the ovarian reserve [27], it can be easily associated with hysteroscopy, which is considered the golden standard for evaluation/treatment of the uterine cavity, and it allows shorter waiting times both for a spontaneous pregnancy and the performance of in vitro fertilization as there are no myometrial incisions or sutures.

Our data show that after the treatment, substantial improvements were achieved in both parameters: the volume and diameter reductions of ablated myomas (73.5% and 37.1% respectively after 6 months) and the quality of life (−84.9% of UFS-QOL Symptom Severity and +44.9% of UFS-QOL total score after 6 months), results that are similar to the ones obtained in previously published studies. Furthermore, we found satisfactory clinical outcomes in terms of operative times and re-intervention rates [28]. This was primarily because 74 myomas out of 106 (69.8%) were ablated transvaginally and hysteroscopically as it was possible to associate different approaches in a single intervention. Vaginal Ultrasound-guided and Hysteroscopic-RFM can be performed under unconscious sedation or spinal anesthesia, have shorter intervention durations, allow earlier hospital discharge and next day return to normal activities. On discharge, all patients received verbal and written instructions and were warned of any symptoms that might be experienced. Additionally, with our protocol there were no fixed times or temperatures of ablation since the treatment of each single fibroid was concluded when the intra-operative CEUS showed an echo-enhanced myoma. In more complicated cases, this can be (and was) achieved with the help of the virtual needle tracking system. This novel approach minimizes both incomplete ablation and over-treatment that can lead to either re-interventions or side effects. Our data introduce elements of standardization, security and technology in the use of RFM; following this new work methodology, we are optimistic about the fact that future literature of this field will achieve greater uniformity in terms of clinical results. The minimally invasive approach of RFM with minimal incision and possible combined approach may result in a rapid and practical development of this surgical technique.

## 5. Conclusions

In conclusion, radiofrequency can be considered a minimally invasive and safe method for the treatment of uterine myomas through the customization and possible combination of transvaginal, laparoscopic or hysteroscopic accesses ensuring less pain, fewer complications and earlier recovery. The standardization of the ablation technique with pre-intervention biopsy and new technology such as virtual needle tracking system (VNTS) and CEUS spares healthy uterine tissue and may change the future management of symptomatic uterine fibroids.

## Figures and Tables

**Table 1:** Baseline characteristics of the study population (*n* = 54).

Age; Mean (Range)	43 (30–62)
UFS-QOL symptom severity score; mean (range)	38.9 (11.8–75.0)
UFS-QOL total score; mean (range)	67.2 (35.3–90.5)
VAS; Mean (range)	4.4 (0–8)
Symptoms; *n* (%)	
menorrhagia	38 (70.4%)
dysmenorrhea	32 (59.3%)
dyspareunia	16 (29.6%)
pollakiuria	6 (11.1%)
Fibroid treated; *n*	106
Fibroid per patient; mean (range)	2.0 (1–4)
Diameter of target fibroids (cm)	3.4 (1.2–7.7)
Fibroid volume (cm^3^)	35.4 (0.9–217.5)
Localization, *n* (%)	
Subserosal (G5–6)	52 (49.1%)
Intramural (G3–4)	44 (41.5%)
Submucosal (G2)	10 (9.4%)
Surgery type, *n* (%)	
transvaginal	64 (60.4%)
laparoscopic	32 (30.2%)
hysteroscopic	10 (9.4%)
Overall surgery time (min)	48 (25–95)
Surgery time (min), by type of surgery:	
transvaginal	23 (10–50)
laparoscopic	35 (20–55)
hysteroscopic	34 (15–40)

VAS = Visual analogue scale; UFS-QOL = Uterine Fibroid Symptom and Quality of Life Questionnaire.

**Table 2:** Improvement in outcomes through follow-up.

	Mean at Baseline (95%CI)	Percentage Variation (95%CI) at 1 Month	Percentage Variation (95%CI) at 12 Months	*p* Value
Fibroid volume (cm^3^)	35.4 (22.0–48.8)	−51.3% (37.3–62.2)	−70.7 (59.9–78.9)	<0.001
Fibroid diameter (cm)	3.44 (3.0–3.9)	−20.1% (12.5–27.1)	−37.1% (29.5–43.8)	<0.001
VAS	4.4 (3.1–5.7)	−45.3% (26.0–59.5)	−74.3% (63.1–82.1)	<0.001
UFS-QOL Symptom Severity Score	38.9 (27.3–50.5)	−84.9% (76.2–90.4)	−74.3% (49.6–86.9)	<0.001
UFS-QOL total score	67.2 (57.7–76.8)	+38.2% (19.9–59.2)	+44.9% (17.1–79.3)	<0.001
Effects of time have been adjusted for age and number of total fibroids for patient.				

## Supplementary Materials

The following are available online at https://www.mdpi.com/1010-660X/56/3/122/s1, Supplement document 1: Uterine Fibroid Symptom & Health-Related Quality of Life Questionnaire (Ufs-Qol).
