# Supplementary material for: A Prospective Intervention Trial on Tailored Radiofrequency Ablation of Uterine Myomas
Source: Medicina (Kaunas). 2020 Mar 12;56(3):122. doi: 10.3390/medicina56030122 (PMC7143923; doi:10.3390/medicina56030122)
Supplement: Supplementary file 1 [file medicina-56-00122-s001.pdf]

# UTERINE FIBROID SYMPTOM & HEALTH-RELATED QUALITY OF LIFE QUESTIONNAIRE (UFS-QOL)

Time since fibroid treatment:

☐ No treatment yet ☐ 3 months ☐ 6 months ☐ 9 months
☐ 1 year ☐ 2 years ☐ 3 years

Listed below are symptoms experienced by women who have uterine fibroids. Please consider each symptom as it relates to your uterine fibroids or menstrual cycle. Each question asks how much distress you have experienced from each symptom during the previous three months.

There are no right or wrong answers. Please be sure to answer every question by checking the most appropriate box. If a question does not apply to you, please mark “not at all” as a response.

During the previous three months, how distressed were you by:

| | Not at all 1 | A little bit 2 | Somewhat 3 | A great deal 4 | A very great deal 5 |
|---|---|---|---|---|---|
| 1. Heavy bleeding during your menstrual period | ☐ | ☐ | ☐ | ☐ | ☐ |
| 2. Passing blood clots during your menstrual period | ☐ | ☐ | ☐ | ☐ | ☐ |
| 3. Fluctuation in the duration of your menstrual period | ☐ | ☐ | ☐ | ☐ | ☐ |
| 4. Fluctuation in the length of your monthly cycle compared to your previous cycles | ☐ | ☐ | ☐ | ☐ | ☐ |
| 5. Feeling tightness or pressure in your pelvic area | ☐ | ☐ | ☐ | ☐ | ☐ |
| 6. Frequent urination during the daytime hours | ☐ | ☐ | ☐ | ☐ | ☐ |
| 7. Frequent nighttime urination | ☐ | ☐ | ☐ | ☐ | ☐ |
| 8. Feeling fatigued | ☐ | ☐ | ☐ | ☐ | ☐ |

The following questions ask about your feelings and experiences regarding the impact of uterine fibroid symptoms on your life. Please consider each question as it relates to your experiences with uterine fibroids during the previous three months.

There are no right or wrong answers. Please be sure to answer every question by checking the most appropriate box. If a question does not apply to you, please mark “none of the time” as your option.

During the previous three months, how often have your symptoms related to uterine fibroids?

| | None of the time 1 | A little of the time 2 | Some of the time 3 | Most of the time 4 | All of the time 5 |
|---|---|---|---|---|---|
| 9. Made you feel anxious about the unpredictable onset or duration of your periods? | ☐ | ☐ | ☐ | ☐ | ☐ |
| 10. Made you feel anxious about traveling? | ☐ | ☐ | ☐ | ☐ | ☐ |
| 11. Interfered with your physical activities | ☐ | ☐ | ☐ | ☐ | ☐ |
| 12. Caused you to feel tired or worn out? | ☐ | ☐ | ☐ | ☐ | ☐ |
| 13. Made you decrease the amount of time you spent on exercise or other physical activities? | ☐ | ☐ | ☐ | ☐ | ☐ |
| 14. Made you feel as if you are not in control of your life? | ☐ | ☐ | ☐ | ☐ | ☐ |
| 15. Made you concerned about soiling underclothes? | ☐ | ☐ | ☐ | ☐ | ☐ |
| 16. Made you less productive? | ☐ | ☐ | ☐ | ☐ | ☐ |
| 17. Caused you to feel drowsy or sleepy during the day? | ☐ | ☐ | ☐ | ☐ | ☐ |
| 18. Made you feel self-conscious of weight gain? | ☐ | ☐ | ☐ | ☐ | ☐ |
| 19. Made you feel that it was difficult to carry out your usual activities? | ☐ | ☐ | ☐ | ☐ | ☐ |
| 20. Interfered with your social activities? | ☐ | ☐ | ☐ | ☐ | ☐ |
| 21. Made you feel conscious about the size and appearance of your stomach? | ☐ | ☐ | ☐ | ☐ | ☐ |
| 22. Made you concerned about soiling bed linen? | ☐ | ☐ | ☐ | ☐ | ☐ |
| 23. Made you feel sad, discouraged or hopeless? | ☐ | ☐ | ☐ | ☐ | ☐ |

During the previous three months, how often have your symptoms related to uterine fibroids?

| | None of the time 1 | A little of the time 2 | Some of the time 3 | Most of the time 4 | All of the time 5 |
|---|---|---|---|---|---|
| 24. Made you feel down-hearted and blue? | ☐ | ☐ | ☐ | ☐ | ☐ |
| 25. Made you feel wiped out? | ☐ | ☐ | ☐ | ☐ | ☐ |
| 26. Caused you to be concerned or worried about your health? | ☐ | ☐ | ☐ | ☐ | ☐ |
| 27. Caused you to plan activities more carefully? | ☐ | ☐ | ☐ | ☐ | ☐ |
| 28. Made you feel inconvenienced always carrying extra pads, tampons, and clothing to avoid accidents? | ☐ | ☐ | ☐ | ☐ | ☐ |
| 29. Caused you embarrassment? | ☐ | ☐ | ☐ | ☐ | ☐ |
| 30. Made you feel uncertain about your future? | ☐ | ☐ | ☐ | ☐ | ☐ |
| 31. Made you feel irritable? | ☐ | ☐ | ☐ | ☐ | ☐ |
| 32. Affected the size of clothing you wear during your periods? | ☐ | ☐ | ☐ | ☐ | ☐ |
| 33. Made you feel that you are not in control of your health? | ☐ | ☐ | ☐ | ☐ | ☐ |
| 34. Made you feel weak as if energy was drained from your body? | ☐ | ☐ | ☐ | ☐ | ☐ |
| 35. Made you concerned about soiling outer clothes? | ☐ | ☐ | ☐ | ☐ | ☐ |
| 36. Diminished your sexual desire? | ☐ | ☐ | ☐ | ☐ | ☐ |
| 37. Caused you to avoid sexual relations? | ☐ | ☐ | ☐ | ☐ | ☐ |

For each symptom indicate on the Visual Analogue Scale the mean intensity:

Menometrorrhagia

0 ________________________________________________ 10

Dysmenorrhea

0 ________________________________________________________ 10

Dyspareunia

0 ________________________________________________________ 10

Pollakiuria

0 ________________________________________________________ 10
